# On the Evidence of Thermodynamic Self-Organization during Fatigue: A Review

**DOI:** 10.3390/e22030372

**Published:** 2020-03-24

**Authors:** Mehdi Naderi

**Affiliations:** 1 Technical Data Analysis Inc., Falls Church, VA 22042, USA; mnaderi@tda-i.com or mnaderi@gwu.edu; 2 Department of Mechanical and Aerospace Engineering, George Washington University, Washington, DC 20052, USA

**Keywords:** self-organization, non-equilibrium thermodynamics, fatigue, persistent slip bands, dislocations

## Abstract

In this review paper, the evidence and application of thermodynamic self-organization are reviewed for metals typically with single crystals subjected to cyclic loading. The theory of self-organization in thermodynamic processes far from equilibrium is a cutting-edge theme for the development of a new generation of materials. It could be interpreted as the formation of globally coherent patterns, configurations and orderliness through local interactivities by “cascade evolution of dissipative structures”. Non-equilibrium thermodynamics, entropy, and dissipative structures connected to self-organization phenomenon (patterning, orderliness) are briefly discussed. Some example evidences are reviewed in detail to show how thermodynamics self-organization can emerge from a non-equilibrium process; fatigue. Evidences including dislocation density evolution, stored energy, temperature, and acoustic signals can be considered as the signature of self-organization. Most of the attention is given to relate an analogy between persistent slip bands (PSBs) and self-organization in metals with single crystals. Some aspects of the stability of dislocations during fatigue of single crystals are discussed using the formulation of excess entropy generation.

## 1. Introduction

Non-equilibrium thermodynamic field of research has been interestingly important with the nowadays motivation for productive and revolutionized materials. Through the new era of technologies, manufacturing and fabrication of materials go beyond the traditional methods. Thereby, there are possibilities to escape “the tyranny of equilibrium phase diagram” [1,2] and to produce materials with highly non-equilibrium conditions and engineered self-organized structures. If we understand the physics of self-organization, dissipative structures, fluctuations, order/disorder, and instabilities, we may be able to induce more controlled and practical self-organization process via externally managed fields [3].

The term self-organization was first introduced by cybernetician William Ross Ashby in 1947. Later, the principle of “order from noise” was formulated in 1960 by cybernetician Heinz von Foerster who stated that random noise and fluctuations facilitate the process of self-organization and open up a verity of states for the system under process. Nobel Prize winner Ilya Prigogine then adopted the notion of self-organization to describe thermodynamic processes far from equilibrium [4,5,6,7]. He showed that for the processes far from equilibrium how amplified small fluctuations can create patterns, orderliness, and novel phenomena. According to Prigogine self-organization is used to explain the ability of non-equilibrium systems evolving in either time or space to spontaneously develop dissipative structures, patterning and orderliness at a critical phase transition between order and chaos. Around the same time, Haken suggested the concept of synergetics and investigated the formation of patterns and structures in open systems far from thermodynamic equilibrium [8]. Later, self-organized criticality (SOC) concept was introduced by Bak et al. to describe a dynamic organizing process with different spatiotemporal growth naturally evolving toward a critical state [9]. The key feature in Bak et al. [9] work is that the system is marginally stable against perturbation. Therefore, self-organization is a process of pattern formation at the higher level emerging from interactions among the lower-level components of a system far from equilibrium [10].

The generic characteristics of the occurrence of thermodynamic self-organization are a) far from equilibrium process, b) non-linear behavior, c) open system with exchanging matter and energy, d) complex (at least two independent variables), d) observable on higher length scale (i.e., macroscale), e) spontaneous. The notion of self-organization of thermodynamic systems far from equilibrium has been vastly used in many scientific research areas especially material science.

The evidence of self-organization is ubiquitous in either natural or man-made systems. It is not possible to cover all of them. For the purpose of this review, thus, only a limited portion of metallic components with specific crystal structure subjected to cyclic loading is the target of this review. Readers interested in this topic are referred to Walgraef [1], Ghoneim and Walgraef [11], Toten and Fax-Rabinovich [12], and related impressive published articles for some of the detailed applications of self-organization in the material science. It is noted that only some representative references related to thermodynamic self-organization are listed in this review. Nevertheless, the research articles pertained to the concepts of thermodynamic self-organization, pattern formation, and dissipative structures in material science are huge in number and astonishing in the content. Some brief examples of evidence and applications of self-organization are:

(a) Development of materials with multielement: Microstructure of materials (i.e., chemical composition, atomic arrangements, crystallographic orientation, precipitants, and defects) manipulated from thermo-chemical and thermo-mechanical processes governs physico-chemical–mechanical properties, specifications, and behavior. Within the emergence of new manufacturing and processing technologies, the possibilities of developing materials far from thermodynamic equilibrium with novel and unusual properties absent under the equilibrium phase diagram are not far-fetched. A rich variety of microstructures and phases is offered in the design of multielement material (i.e., multielement alloys, high entropy alloys) with desirable performance characteristics. Significant signs of progress have been achieved in the development of metamaterials and alloys containing more elements such as [13,14,15,16,17,18,19] and [14,20,21,22,23,24,25,26]. Their compositional and microstructural vastness has opened serious technical challenges in the material science community.

(b) Development of wear resistance materials, surface engineering, self-cleaning/self-lubricating surfaces: Interaction of two surfaces in relative motion is accompanied by energy dissipation (friction) and material degradation (wear). The volume of wear depends on the severity of friction and many factors such as applied load, velocity, material properties, and size of the contact area. Friction as an irreversible non-equilibrium process can be explained based on the concepts of non-equilibrium thermodynamics and self-organization [12]. The primary goal of applying self-organization is to decrease wear intensity and coefficient of friction and to extend the stable wear stage. Therefore, the concept of self-organization in tribosystems, if applied correctly, can provide substantial improvement in the wear characteristics of tribosystems and can result in the self-protection of frictional surfaces [12]. In addition, the ultimate benefits such as tool’s life improvement, high quality surface finish and enhanced dimensional accuracy can be attained [12].

Since the pioneering works of Klamecki, Kostetsky, Bushe, Bershadsky [27,28,29,30,31,32,33,34], numerous outstanding scientific works regarding self-organization and its applications in tribology and surface engineering are published in the literature [12,28,30,32,35,36,37,38,39,40,41,42,43,44,45,46,47,48,49,50,51,52,53,54,55,56,57,58,59]. A comprehensive discussion and detailed examples of self-organization during friction are given by Fox-Rabinovich and Totten [12], and Nosonovsky and Mortazavi [60]. Process of self-organization occurrence in tribosystems can be concluded if friction coefficient, and wear rate are reduced [12]. Examples of self-organization and patterning in tribosystems could be the formation of tribo-films (or “secondary structures”), stick-slip motion, and mutual adjustment of contact bodies [12,60]. Friction coefficient, wear rate, or Shannon entropy might be a measure of self-organization occurrence in contact bodies [12,38,60]. It is noted that the formation of dissipative structures during running-in period and their stability during post running-in stage (steady stage) can slow down the wear rate [12]. Therefore, through the notion of self-organization, novel high-wear resistance materials are possible.

(c) Hierarchically self-organized particles in polymers: Another research field where pattern formation, self-organization process, and dissipative structures play a vital role is polymer and nanocomposite research field. Some selective research works are in Refs. [61,62,63,64,65,66,67,68,69,70]. Some of the applications of self-organized nanoparticles are the development of polymeric materials with improved properties in electromagnetic, catalytical, and optical fields. Additionally, the use of rubber/nanoparticle composite in car tires having both the viscoelastic properties of soft rubbers and the hard characteristics of nanofillers particles is increasingly demanding. As an example, Hashimoto and co-workers investigated the process of self-organization and dissipative structures formation in polymer melts with different kinds of dispersed nanoparticles [61,62,63,64]. They used two types of fillers (silica and carbon black) and two types of cross-linked poly (styrene-ran-butadiene) rubbers (SBR) to prepare four kinds of the fillers/rubbers composites. The goal was to elucidate the influences of polymer/filler interplays on the formation of self-organized dissipative structures subjected to a processing condition. The hierarchical and “cascade evolution of the dissipative structures” [69] were developed through the thermal fluctuations at non-equilibrium conditions. According to Hashimoto and co-workers, initially the nano-particles were fused together into the aggregates followed by the formation of clusters composed of the Si- or carbon black (CB)-based aggregates bound together by the cured SBR matrix. Fractal agglomerates were then developed and dispersed within a cured SBR matrix. The final stage was the bulk composite with dispersed fractal agglomerates. The rubber/filler composites, with the viscoelastic characteristics of soft rubbers and the hard properties of nano-particles have been increasingly demanded for applications such as economic automotive tires.

In another work by Hashimoto et al. [62], the filler particles were carbon black (CB) dispersed in two polymers called SBR and polyisoprene (PI). According to them, the process of cross-linking in matrix polymers lock self-organized particle-based dissipative structures at non-equilibrium state over the length scale from ~0.1 nm to ~20 μm. Using a combined scattering technique in three-dimensional space, they were able to observe dissipative structures formation. The authors argued that the self-organization of mass-fractal agglomerates of filler particles into polymer matrices starts with the fracture of filler particles to bare clusters and their flow into polymer matrices due to induced mechanical work of mixing.

Among other numerous application fields, we can name laser-induced irradiation phase transformation fields [3,11,71,72,73]. The fundamental concepts such as non-equilibrium thermodynamics, entropy, the process of self-organization, formation of dissipative structures, and thermodynamic stability analysis provide a powerful tool allowing tangible practical benefits on the development of novel materials with exceptional properties. Detailed overview of self-organization related examples help to bridge the gap between theory and practical motivation.

The rest of the review is organized as follows. First, a brief review on irreversible thermodynamics and entropy are given. Afterward, the analogy of self-organization and fatigue of metallic structures are discussed with evidence taken from the literature followed by discussion related to the stability of dislocations (especially single crystals) using the formulation of excess entropy generation. The review is finally concluded.

## 2. Theoretical Overview of Irreversible Thermodynamics and Entropy

**Figure 1:**
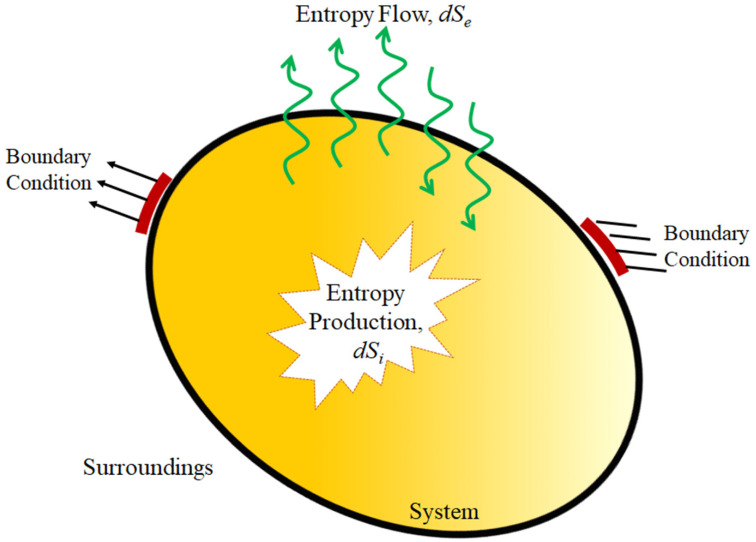
Entropy exchange in an open system. *dS_e_* is entropy flow through the boundaries of the open system and *dS_i_* is the entropy production within the open system.

The majority of the processes surrounding us are irreversible processes at non-equilibrium conditions. Their evolutions are spatiotemporal with at least one non-vanishing flux. Contrary to non-equilibrium states, at a thermodynamic equilibrium state, the flow of energy and matter vanishes, entropy of the system is at the highest level, and entropy production disappears. For an open system interacting with the environment, formulating total entropy exchanges are required to elucidate the irreversible processes. Irreversible thermodynamics developed by e.g., Onsager [74,75], and Prigogine [7] can describe systems away from the equilibrium state. The change of total entropy of an open system exchanging energy and matter with surrounding consists of two parts including a flow of entropy due to the interaction with the environment, and entropy production due to the processes taking place within the system [4].

(1) $$dS=dS_{e}+dS_{i}$$

where $dS_{e}$ and $dS_{i}$ are the entropy exchange with surroundings and the entropy produced internally by the system, respectively, as shown in Figure 1. While entropy flow can be positive, negative, or zero, entropy production is always equal (for reversible process) or greater (irreversible process) than zero according to the second law of thermodynamics.

The mechanisms of self-organization necessitate interactions at different length and time scales. Across different length scales (nano, molecular, micro, meso, and macro), while overall entropy generation is positive to confirm second law, at smaller scales, the rate of entropy generation fluctuates around positive and negative values. The decrease in the entropy at one scale might be compensated by the entropy increase at another scale [76]. Hence, a hierarchical approach to entropy production might be needed for multiscale analysis of the thermodynamic process. The net entropy production can be written as the sum of entropy production at different length scales [40,76,77].

(2) $$dS_{i}=dS_{i}^{nano}+dS_{i}^{micro}+dS_{i}^{macro}$$

For example, as stated by Nosonovsky and Esche [77], thermally activated grain growth in metals is a self-organization process in which multiple length scales are involved. At the macroscale, entropy change is zero ($dS_{i}^{macro}=0$). At microscale (scale related to the grain sizes), entropy decrease is associated with grain growth and more orderliness. However, at the nanolevel, energy barriers for the grain growth must be prevailed due to the random thermal fluctuation of atoms resulting in nanoscale entropy increase [77]. Thus, the decrease in the entropy at the microscale is compensated by an increase in the entropy at the nanoscale. This example shows that for some irreversible processes such as fatigue degradation, while the bulk material goes through a reversible process with zero entropy generation, at lower length scales (i.e., micro and nano scales) it might be involved in the irreversible process that is not observable at the macro level [76].

Within the framework of statistical physics and using fluctuation theorem, it is established that as the length or time scales becomes very small, entropy generation might become negative on very short time or length scale. While entropy generation grows on average to be compatible with the second law, at a very small spatial and temporal scales entropy generation fluctuates and might become negative [78,79,80,81,82,83,84,85]. Ostoja-Starzewski and Malyarenko [78] showed that the irreversible entropy is a submartingale with the sum of a martingale (M) and an increasing process (G). Therefore, for an irreversible process, M is unequal to zero and G is greater than zero with randomly occurring negative entropy generation.

Using the assumption of local equilibrium, Equation (1) can be written in a volumetric form [2,4,7].

(3) $$\frac{ds}{dt}+\nabla .J_{e}=\sigma$$

where *s* is the density of total entropy, *J_e_* is the flux of entropy per area per time, and *σ* is the density of entropy production rate as follows.

(4) $$\sigma =\frac{ds_{i}}{dt}\geq 0$$

(5) $$-\nabla .J_{e}=\frac{ds_{e}}{dt}$$

with *s_e_* and *s_i_* as the volumetric entropy flow and production.

For Irreversible processes, entropy production can be written in terms of thermodynamic forces (*X_i_*) and thermodynamic flows (*J_i_*) as follows.

(6) $$\sigma =\frac{ds_{i}}{dt}=\sum^{\;}X_{i}J_{i}$$

It is noted that the above statements for the second law of thermodynamics are derived based on the notion of local equilibrium. For systems at non-equilibrium conditions, thermodynamic variables such as temperature, pressure, and concentration can be locally defined over an elemental volume which is locally at equilibrium [2]. Length scale and timescale in the local equilibrium framework are important parameters which need more clarifications. According to the local equilibrium assumption, a thermodynamic system which is globally at non-equilibrium condition, can be divided into elements. In terms of length scale, the elements are large enough to be immune against fluctuations, albeit sufficiently small for the validity of equilibrium assumption [86]. In terms of time scale, the time required for the evolution of variables within systems (i.e., systems with microscopic, mesoscopic or macroscopic length scale) is much larger than the time required for two successive collisions between particles [86]. It is noted that the local equilibrium assumption is an approximation [2] which may be justified for the systems considered in this review. However, for systems with the fast thermodynamic processes such as nuclear collisions, shock waves, and ultrasounds in which the timescale is very short and large gradient involves, the assumption of local equilibrium notion might be questionable [2,86].

Among all non-equilibrium conditions, the stationary states are particular cases and have privilege due to their continuous existence without change for a sufficiently long time [2,4,7]. For non-equilibrium systems at stationary, total entropy is constant meaning that produced entropy must be pumped out of the system [4]. Then, at steady states, Equation (3) can be written as:

(7) $$\sigma -\nabla .J_{e}=0$$

According to Equation (7), to keep non-equilibrium system at steady states, the required condition is to continuously pump negative entropy flux with the same magnitude as entropy production to the surrounding [4].

### 2.1. Linear Non-Equilibrium Region; Near Equilibrium

As shown in Equation (6), volumetric entropy production is quantified through thermodynamic forces and thermodynamic flows for irreversible processes either close or far from equilibrium. For systems close to equilibrium, the thermodynamic forces and flow are linearly related through some phenomenological coefficients (L_kj_) as follows [2].

(8) $$J_{k}=\sum^{\;}L_{kj}X_{j}$$

where coefficients L_kk_ characterize for example heat conductivity, electric conductivity, chemical affinity. Coefficients L_kj_ (k≠j) represent the interactions between two irreversible processes such as thermoelectric, thermodiffusion, and electrodiffusion. While coefficients L_kk_ should be positive, coefficients L_kj_ (k≠j) can have either positive or negative sign obeying Onsager reciprocal relations (L_kj_ = L_jk_).

Prigogine demonstrated that non-equilibrium systems within a linear regime evolve to the stationary states with minimum entropy production [4,7]. Linear non-equilibrium systems which cannot come to equilibrium, attempts to evolve toward a minimum level of entropy production. Prigogine’s minimum entropy production principle is valid within the requirements of a) time-independent boundary conditions, b) linear phenomenological law, c) constant and symmetric phenomenological coefficients [86]. Any perturbation in the thermodynamic system near equilibrium with a linear response is regressed and the irreversible process drives the system back to the undisturbed condition to be compatible with Prigogine’s minimum entropy production principle [2]. The positivity of entropy production and negativity of time variation of entropy production ensure the stability of linear non-equilibrium stationary states [2,4].

### 2.2. Non-Linear Non-Equilibrium Region; Far from Equilibrium

In reality, thermodynamic systems are subjected to perturbations raised either externally from surroundings or internally from random fluctuations generated by the systems. Multiple solutions and possibilities only emerge from systems with non-linear behavior. Hence, multiple and unexpected solutions during linear irreversible processes are automatically ruled out. In the linear non-equilibrium regime, fluctuations are damped and the system returns back to the minimum entropy production level. While close to equilibrium, fluctuations are damped in the neighborhood of the fixed point, far from equilibrium and beyond a certain threshold, oscillations are no longer damped out but maybe amplified. Far from equilibrium nonlinear regime, perturbations and the law of probability govern the system fate. In far from equilibrium, the system makes choices among a few possible solutions. Self-organization is one of the possibilities that the system makes [2,4].

A thermodynamic system at highly non-equilibrium condition (far from equilibrium condition) is more active and structural elements work consistently, and fluctuations synergistically occur. At the critical moment, new structures are created and the thermodynamic system transfer to a new condition [4,5]. Entropy production during nonlinear non-equilibrium processes with highly ordered dissipative structures is lower than entropy production before self-organization [87,88].

According to Prigogine [4,5,6,7], non-equilibrium processes might be a source of order and might lead to a new state of matter called dissipative structures. Far from equilibrium energy and matter transfers (i.e., heat, wear, dislocation generation) which were considered a source of waste in classical thermodynamics become a source of order. Thermodynamic processes far from equilibrium may bring order into the system using self-organization and dissipative structures [5,6]. In this case the non-equilibrium process is a source of order and self-organization is defined as the formation of order and as a process of formation of dissipative structures. Dissipative structures are the results of fluctuations which are not stable with a small deviation from equilibrium. The results of self-organization of dissipative structures are negative entropy generation or positive growth of free energy [2,89]. Due to the process of self-organization, dissipative structures allow the system to reduce the internal entropy generation and counteract with the deterioration process [90].

As a result of patterning and self-organization, the newly ordered thermodynamic system reaches to the lower entropy production compared to the thermodynamic system before the occurrence of dissipative structures [47]. Prigogine’s theory of minimum entropy production is formulated and applied by [47,87,88] during the processes of self-organization. Far from equilibrium, thermodynamic flows are no longer a linear function of thermodynamic forces. The stability of far from equilibrium nonlinear systems is fully governed by fluctuations and the law of chance [91]. It is not feasible to establish a criterion for the occurrence of self-organization in a dissipative process. However, one can establish a criterion based on the loss of thermodynamic stability as an indication for self-organization occurrence and appearance of patterned structures [35]. According to Prigogine, thermodynamic stability can be determined using the sign of the second variation of entropy production (Lyapunov’s function) [4,5,6].

(9) $$\delta^{2}s_{i}<0$$

The stability condition is

(10) $$\frac{\partial \left(\delta^{2}s_{i}\right)}{\partial t}\geq 0$$

Writing entropy production in terms of thermodynamic force (X) and flow (J):

(11) $$\frac{1}{2}\frac{\partial \left(\delta^{2}s_{i}\right)}{\partial t}=\sum^{\;}\delta X_{n}\delta J_{n}$$

with $\delta X_{n}\;\mathrm{and}\;\delta J_{n}$ as the deviation of thermodynamic force and flux from a stable state. They are known as excess entropy generation terms. Examples of thermodynamic fluxes and forces are heat flux and temperature gradient; gradient of chemical potentials and diffusion; the difference in chemical affinity and chemical reactions; gradient of mechanical stresses and deformation.

The Equations (9)–(11) are very important to extend thermodynamics to non-equilibrium processes in terms of an explicit expression for entropy production and demonstrate the most energetically favorable deviations in the thermodynamic variables. The above expression sheds light on the stability of a process far from equilibrium by studying excess entropy term and fluctuation of thermodynamic force and flux.

## 3. Self-Organization Evidence during Fatigue

In this section, the author aims to first elaborate the formation of dissipative structures leading to patterning or self-organization phenomenon during fatigue degradation of metals, especially with single-crystal structures. It is noted that by the “self-organization” or “self-organized” term frequently used during this review, the author means orderliness and patterning in dissipative structures. Only a few of the evidences taken from impressive works available in the literature are reviewed to show the self-organization signatures during fatigue degradation.

Fatigue as a non-equilibrium process can be viewed according to concepts of non-equilibrium thermodynamics, entropy generation, and self-organization. The process of plastic deformation is highly non-linear, dissipative, and far from equilibrium. Plastic deformation is usually a detrimental non-equilibrium process leading to fracture and failure. During cyclic plastic deformation, the process of dislocations/defects/faults pill-up, rearrangement, movement, multiplication, and annihilation eventually lead to the nucleation and formation of micro/macro fatigue cracks. However, in some cases, cyclic plastic deformation may be beneficial and trigger self-organized microstructures formation. In crystal structures in which for example persistent slip bands (PSBs) form during cyclic deformation, studying the connection among fatigue crack initiation, cyclic plastic strain localization, PSBs formation, and self-organization are of paramount importance to design fatigue resistance and durable material. For fatigue degradation of the metallic part, the attention is mostly given to the works available in the literature pertaining to face-centered cubic (FCC) single crystals (i.e., copper, nickel, silver, etc.) and connection between self-organization and PSBs formation.

It is noted that the process of fatigue degradation should be viewed at different length scales. For example, at small scale creation of self-organization might reduce entropy generation. However, at the upper, scale the self-organization occurrence might interpret as the initiation of cracks. As an example, consider high cycle fatigue of metals, at grain scale, the formation of patterned-dislocations may be viewed as configurational entropy which brings entropy generation down [76]. While, the decrease of entropy at the grain level is counteracted by microscale entropy generation through micro cracks formation.

### 3.1. Self-Organization and PSBs Formation

The behavior of a metal structure under cyclic loading is greatly affected by crystals’ orientation, stacking fault energy, dislocation movements (i.e., edge and screw dislocations, cross slips, annihilation) and slip modes. Dislocations’ patterns and arrangements such as dipolar, veins, PSBs, labyrinth, and cells are directly connected to the orientation of crystals and slip modes [92,93,94].

Persistent slip bands (PSBs) formation is considered as a result of spontaneous formation and evolution of localized dissipative structures (i.e., dislocations, veins, slip bands, etc.). The spontaneous formation of dissipative structures is a so-called self-organization process in a thermodynamic system far from equilibrium. Under cyclic loading and above a certain value of the driving forces (or so-called thermodynamic force *X_n_* mentioned previously), thermodynamic instability takes place and PSBs emerge as a result of the transformation to a newly ordered, pattern and periodic structure [95]. The evolution of dislocation structures is regarded as a result of pattern formation and process of self-organization [96,97,98,99,100,101,102,103]. From a macro-scale point of view, the instability point is regarded as the initiation of saturation in cyclic strain-stress response after which the PSBs undergo a stable or quasi-stable regime.

**Figure 2:**
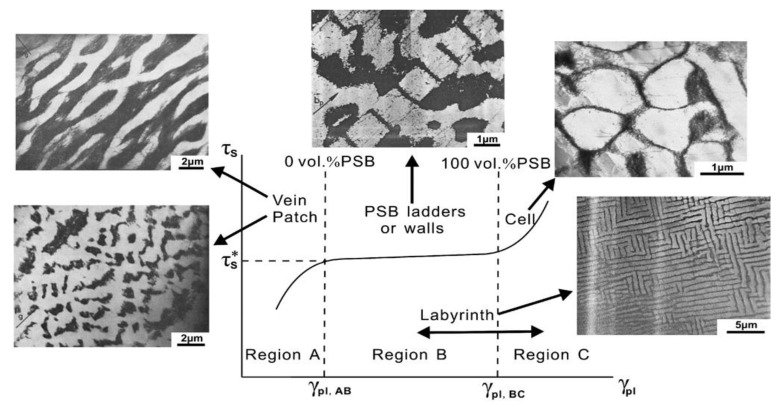
Schematic of the cyclic stress-strain curve along with the evolution of dislocation pattern of copper single crystal for single slip [92] (with permission) (Images on this original figure quoted from [104,108,117,118]).

**Figure 3:**
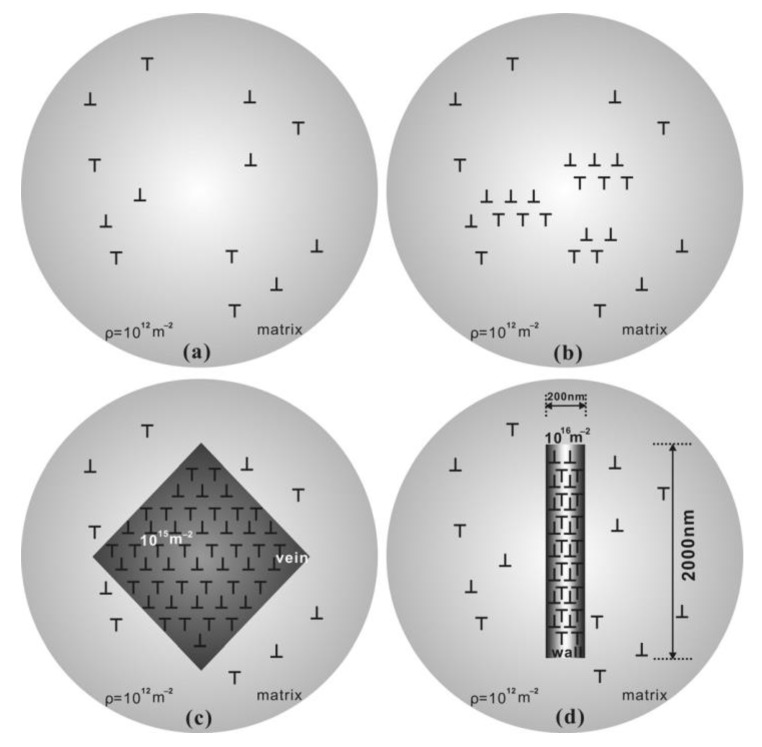
Schematic of macroscopic evolution of dislocation configuration during fatigue: (**a**) dislocation sources; (**b**) dipole segment; (**c**) vein structure; and (**d**) persistent slip band (PSB)-ladder structure [93]. (with permission).

**Figure 4:**
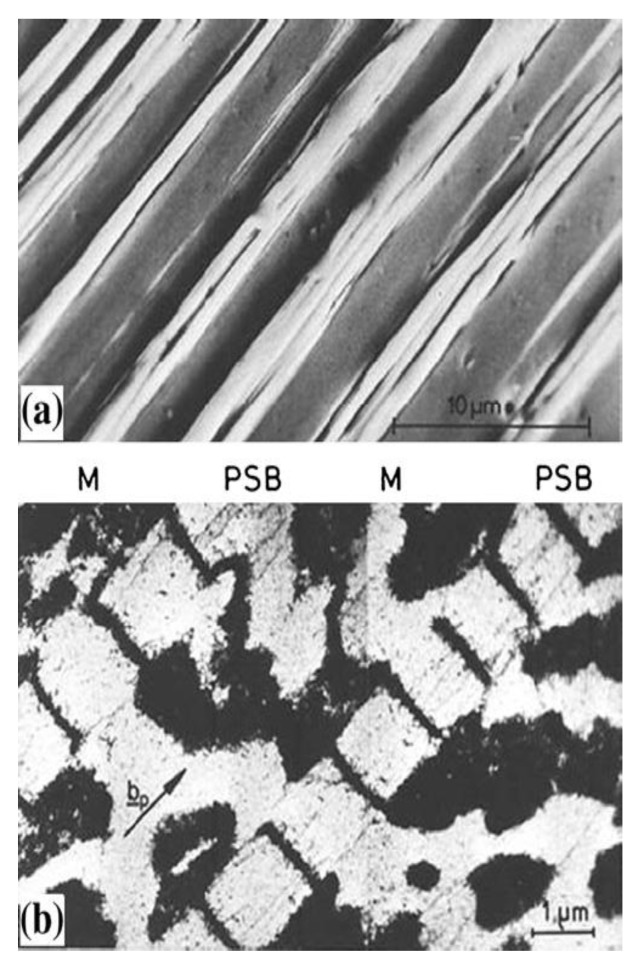
PSBs in fatigued copper; (**a**) SEM of surface; (**b**) transmission electron microscopy (TEM) micrograph of PSBs with ladder structure between matrix of dipolar veins [92,113] (with permission).

**Figure 5:**
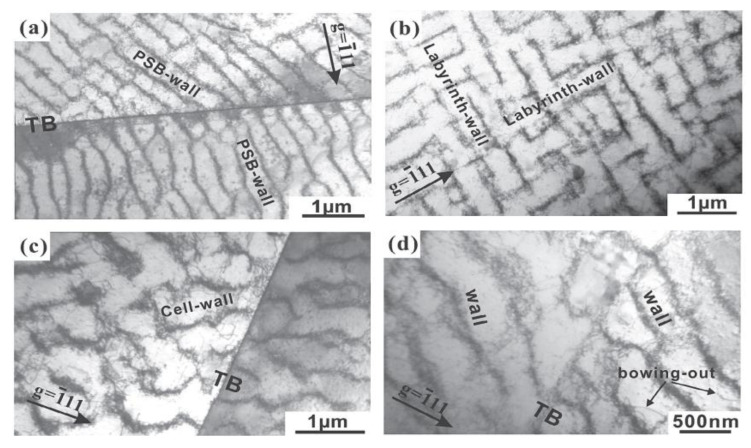
TEM image of dislocation patterns in polycrystalline copper-5%aluminum under cyclic plastic strain of 0.001: (**a**) PSB wall; (**b**) labyrinth; (**c**) cell wall; (**d**) wall structure [115] (with permission).

Experimental cyclic stress-strain (CSS) curve for copper single crystals oriented for single slip at room temperature and under low plastic strain by Mughrabi [104] shows clear three stages of deformation and microstructural evolution. The relationship between microscopic dislocation pattern and macroscopic deformation behavior can be established through understanding cyclic stress-strain (CSS) curve. Figure 2 [92] depicts CSS of copper single crystal and the formation of various dislocation structures for fatigue FCC single crystal. Three distinct phases (A, B, and C) are clear in this figure. As schematically shown in Figure 3 obtained from the comprehensive work of Li et al. [93], FCC crystals initially contain many pre-existing dislocation sources. Once crystal undergoes fatigue loading, these sources emit dislocations with opposite Burger vector attracting each other to form dipoles. Once the distance between opposite dislocations reaches a critical distance, stable multipoles are formed from many dipole segments followed by multiplication and annihilation process [93]. With further cycling, multipoles are collectively aggregate and continuously evolve, followed by spontaneously stable vein structures formation. As fatigue progresses, dislocation density in the veins slowly grows and at a critical density, the annihilation of stored dislocations is dominant resulting disintegration process of veins [97]. Early PSB-ladder structures are appeared by initial instability of dipoles near the center of veins and break-up of veins [105] and the onset of region B of Figure 2 is marked as the first appearance of PSBs ([106,107,108]. In order to achieve a saturation regime, a dynamical equilibrium should be attained between multiplication and annihilation of dislocation structures. For low or intermediate strain amplitude, annihilation and multiplication of dislocations are roughly balanced, otherwise, the balance is not achieved [109]. With further increasing fatigue cycles, the volume fraction of PSBs is increased and extends all over the crystals within sample test. As PSBs are fully developed and with the increasing number of cycles, secondary slips are activated, secondary dislocations formed and saturation regime is ended [109]. That is, crystals undergo an additional transformation into the formation of more complex structures. Termination of the plateau region is marked with the initiation of secondary hardening and formation of cells or labyrinth structures [110,111,112,113,114]. As an example, Figure 4 and Figure 5 show patterned dislocation structures, PSBs, walls, cells, and labyrinth for fatigued copper single crystal and copper-5% aluminum [113,115].

The appearance of patterned and self-organized dislocation structures mentioned above can be divided into different stages of instabilities [97]: 1) First, the aggregation and annihilation of multipoles become unstable and form vein structures [93,97,116]; 2) veins at their centers then break into walls or both veins and walls are developed alongside the slow refinement of veins [93,97,106]; 3) secondary dislocations are formed once PSBs are fully developed and new structural configurations such as cells, labyrinth are formed [109].

It is to be noted that these instabilities might take place at different length/time scales. One should view the process of thermodynamically dislocation evolution as a multi-temporal and spatial phenomenon. For example, at micro-scale, the crystal structure might not show any sign of self-organization, while at one scale down (nano-scale) locally crystals might experience instability and transition from less order structure to more ordered ones (i.e., transition from multidipoles to veins). Therefore, in a non-equilibrium thermodynamic process, self-organization, the formation of patterned structures, and dissipative structures (eventually entropy generation) should be viewed as a multi-scale/temporal process. In addition, dislocations’ structure evolution during fatigue as a non-equilibrium system shows the hierarchical self-organization process. Energy dissipation process from an initial structure with distributed stored dislocations to the onset of crack initiation takes place via a “cascade evolution of the dissipative structures” (dipole, veins, PSBs, labyrinth, cells, etc.).

**Figure 6:**
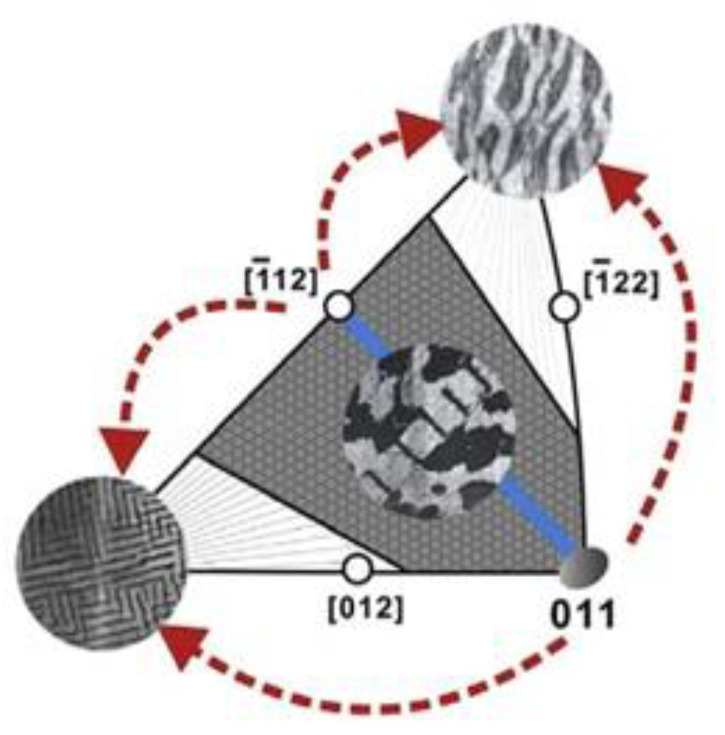
The effect of orientation on the dislocation configurations of face-centered cubic (FCC) single crystals [121] (with permission).

We emphasize that the presented examples are a special case of single crystals oriented for single slip and formation of PSB-ladder structure and appearance of the plateau regime are key features in CSS curve. In addition, it is seen that in i.e., copper single crystals, PSBs formation and their evolution towards a plateau of fully developed PSBs are the results of material instabilities followed by patterning and self-organization process. However, it should be noted that the formation of dislocation configurations, arrangements/patterning, hardening rate, and saturation regime during cyclic loading are greatly influenced by crystal size, orientation, slip modes, stacking fault energy, level of applied cyclic stress/strain amplitude. For a general FCC metal/alloy, formation of PSBs-ladder and emergence of plateau region may not be the case and different formation mechanisms for dislocation arrangements, patterning, aggregation, and evolution are involved. The basic features among all varieties of dislocations arrangements (dipole, vein, patch, PSBs, wall, cell, labyrinth, etc.) are spatial or temporal periodically ordered and self-organized structures [93,119,120]. Figure 6 shows the effect of orientation on cyclic deformation and dislocation configurations as divided into different regions in the stereographic triangle [121,122,123]. It is observed that classical plateau behavior is apparent in the interior of the stereographic triangle single slip oriented copper single crystals. Depending on orientation and slip modes appearance of plateau either exists or rules out [123]. Copper, nickel, and silver crystals exhibit many resemblances in the cyclic deformation behavior. Crystal orientation influence on the dislocation arrangements of FCC single crystals is presented in Figure 6 [121]. Comprehensive discussions are referred to the works of [93,115,119,121,122,123,124,125,126,127,128,129].

### 3.2. Self-Organization and Stored Energy Concept

Inspired from the first law of thermodynamics, during plastic deformation of metals, the dominant part of applied external mechanical work is dissipated in the form of for example heat, and acoustic. The remaining part is stored in the form of dissipative structures (dislocations structures, PSBs, cells, etc.). Therefore, measuring dissipated energy might be regarded as an indication of dislocation evolution during the deformation process. A rich body of research works is devoted to investigate the relationship among stored energy, dislocation arrangements, self-organization process, dissipated heat, and crack nucleation [130,131,132,133,134,135,136,137,138,139,140,141,142,143,144,145,146,147,148,149,150]. Among them, the initial experimental work of Taylor and Quinney [130] of dissipated energy during plastic deformation of pure copper for a wide range of applied strain has been the subject of attention among many researchers.

Influenced by non-equilibrium thermodynamic arguments developed by Prigogine [4,5,6], Seeger argued that the problem of dislocation formation/evolution during plastic deformation can be treated qualitatively by the application of non-equilibrium thermodynamics of irreversible processes in open systems [133,134,135]. The basic feature of Seeger’s work [133,135] is that during the plastic deformation process, the ratio of stored energy to the mechanical work done is a suitable measure for tendency of a thermodynamic system toward self-organization, pattern creation, and dissipative structures formation. Using cyclic plastic stress-strain curve data of Mughrabi [104], he investigated the relationship between stored energy and the formation of dissipative structures. As presented in Section 3.1, PSBs pattern in cyclic loading can be explained by the concept of self-organization and are bulk features of fatigued crystals. Around the beginning of plateau region, PSBs appear and by the end of the plateau they fully extend over the open system (i.e., specimen). Further increase in plastic strain results in the formation of complex new structures and higher stress [104,113].

In the saturation region of a CSS graph, hysteresis loops are cycle independent. Using tests of Mughrabi [104,151], Seeger argued that at the plateau region dislocation density is in overall constant, consequently, the change of the stored energy per cycle pertained to dislocations is small and insignificant. From a thermodynamic point of view, this corresponds to the dissipation of almost all mechanical work during irreversible fatigue process. It is also noted that as deformation proceeds, dislocations arrangement becomes more order and less random [135,152]. As pointed out by Mughrabi, the requirement for CSS to remains fairly steady state is dynamic equilibrium between dislocation multiplication and annihilation within PSBs and forward/backward slide of the same dislocations in the deficiency of dislocation growth and annihilation within matrix [153].

In addition, other researchers used the concept of stored energy as a criterion for fatigue crack nucleation onset. For example, assuming only a small percentage of input work stored in a fatigued crystallographic structure, Chen et al. [132] and Wan et al. [137,138] established a stored energy-based criterion for crack nucleation. Resembling the Griffith surface energy at the microstructural scale, it is the local stored energy governed by the density of dislocation and dissipative structures accountable for crack nucleation. Experiments on several ferritic steel polycrystal bending fatigue samples to identify crack nucleation sites and cycles were performed by Wan et al. [138]. After fatigue cycles are stabilized, a small fraction (5%) of the area of saturated hysteresis loop was the base for energy storage rate per cycle calculation. Using computational crystal plasticity, they calculated geometrical and statistical necessary dislocations as connected to stored energy per cycle. Within the assumption that the rate of stored energy per cycle is constant after saturation is achieved, they concluded that cumulative stored energy reaches a critical value at the onset of crack nucleation. It is seen that their arguments and rationales have a thermodynamic base and one can immediately connect their findings of a material constant for crack nucleation as a direct application of thermodynamic self-organization and dissipative structures formation.

### 3.3. Self-Organization Evidence in Acoustic Signals and Thermography

Similar to stored energy measurement, non-destructive techniques such as acoustic signals and temperature are useful macroscale indicators to evidence patterning and self-organization occurrence in fatigue-induced structural transformations [98,154,155,156,157,158,159,160,161,162,163,164,165,166]. Among abundant and compelling works concerning measuring the degree of degradation through acoustic emission and thermography, a few selective of them are of interest here to elaborate the rationale between the appearance of patterning and macroscale measurement tools [98,154,155]. However self-organization was not directly addressed in some of the aforementioned references, the patterning occurrence can be justified by analogy among dislocations activities, acoustic sound, and temperature evolution in the course of fatigue.

#### 3.3.1. Self-Heating and Thermography

It is known that the process of self-heating is attributed to the movement and activities of dislocations and defects in the material lattice [167,168]. The process of energy dissipation depends on dislocations’ density, mobility, and interactions. In a very fascinating work, Huang et al. investigated cyclic loading and the subsequent fatigue-induced structural transformations with in-situ neutron diffraction and thermal characterization for a single-phase, polycrystal nickel-based alloy [98,154,155]. They used in-situ neutron-diffraction measurements, high-resolution thermocouples, a high-precision extensometer, and transmission-electron-microscopy (TEM) to accurately measure dislocation substructure, lattice strain evolution, heat generation, and displacements. Different stages of structural evolution including initial hardening, softening, and eventual saturation as discussed abundantly before are identifiable from the results of in-situ neutron-diffraction and temperature-evolution measurements. Their results confirm correlation between the thermal behavior of the sample during deformation and in-situ observation of the time-dependent dislocation pattern.

**Figure 7:**
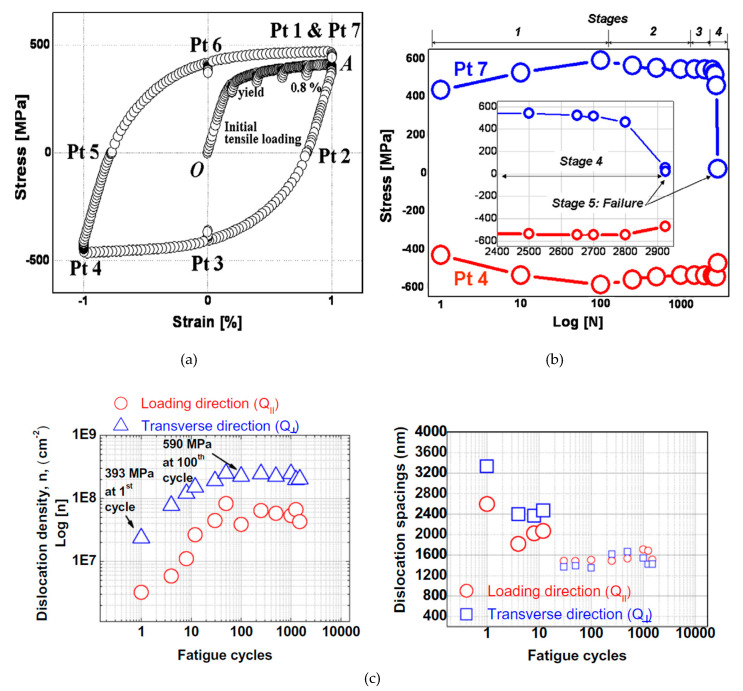
(**a**) hysteresis loop of first fatigue cycle with seven measurement points; (**b**) stress evolution pertained to peak hysteresis loop points of tension (point 7) and compression (point 4); (**c**) dislocation density and dislocation wall spacing as a function of the number of cycles at 1% strain [98,170] (with permission).

Figure 7a depicts the stress-strain loop during one cycle strain-controlled low cycle fatigue test of Huang et al. at the frequency of 1 Hz [98]. They obtained test data at different fatigue cycles ranging from 1 to 2500 for seven points as shown. The plot of stress at the peak tension point (point 7) and peak compression point (point 4) is presented in Figure 7b [98]. Initially, cyclic hardening takes place up to 100 cycles (stage 1) before transitioning to the softening region up to the 1000th (stage 2) accompanying peak stress reduction. At stage 3, stress saturates with a plateau lasting about 1800 cycles after which stage 4 starts with a slight cyclic softening. Final fatigue failure takes place at the 2925th cycle (stage 5). Figure 7b,c show stress, dislocation density, and dislocation spacing driving fatigue process. The results of Figure 7b,c clearly show fatigue induced structural transformation and its connection to dissipative structures, pattern formation, and self-organization process.

**Figure 8:**
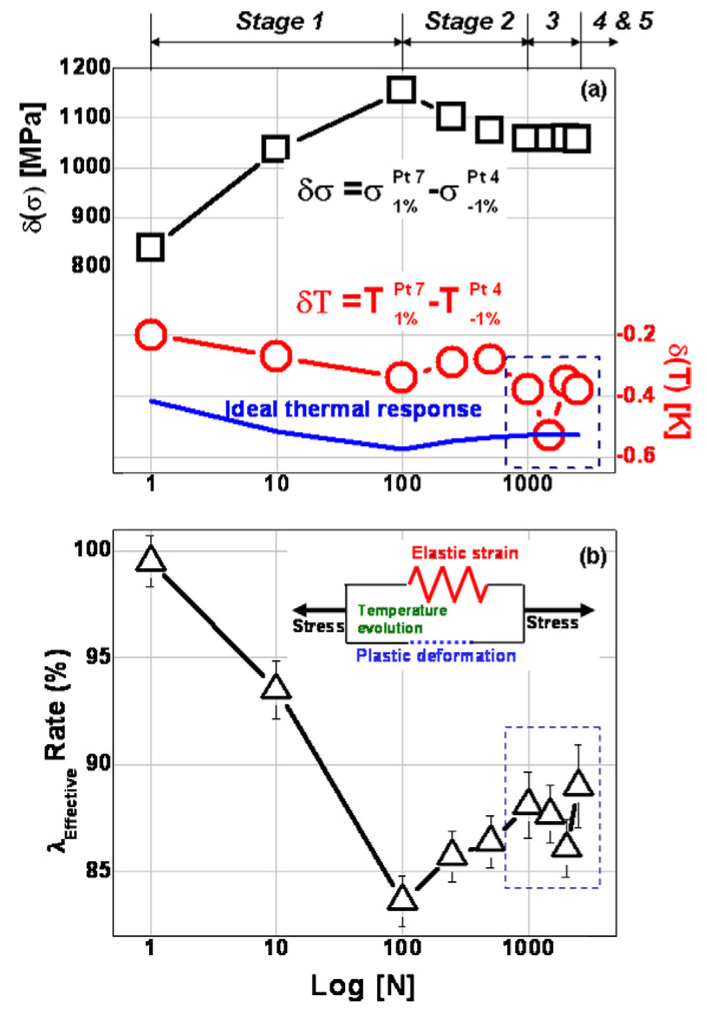
(**a**) The evolution of stress and temperature differences between points 7 and 4 during fatigue life; (**b**) effective heat conduction rate versus fatigue cycles; comparison of the ratio of stored energy percentage and stress evolution during fatigue. (**a**,**b**) are obtained from [98] (with permission).

In order to show the correlation between stress and temperature evolution, Huang et al. plotted differential stress and temperature between points 7 and 4 as presented in Figure 8a. Temperature and stress correlate well up to stage 3 in which stress shows plateau while temperature has a slight change. This anomaly in temperature change in stage 3 and afterward can be explained using an effective-heat-conduction rate ($\lambda_{effective}$) as follows [98].

(12) $$\lambda_{effective}=\frac{Q_{x}^{Pt6}}{Q_{x}^{0}}\times 100$$

where $Q_{x}^{Pt6}$ and $Q_{x}^{0}$ are heat-transfer rates calculated by thermocouple based temperature measurement before and during the cyclic loading for point 6 of Figure 7a, respectively.

The evolution of $\lambda_{effective}$ (Figure 8b) can be explained with Ashby’s conclusions of the thermal conductivity for the metals [169].

(13) $$\lambda_{}=\frac{1}{3}\times C_{e}\times \underline{c}\times l$$

where $C_{e}$ represents the electron specific heat per volume, $\underline{c}$ is electron velocity, and l is mean free path pertained to the microstructure. It is noted that defects (i.e., dislocations) tend to reduce electron mean free path l and consequently λ [98,155].

As presented in Figure 7c, the evolution of the effective-heat-conduction rate ($\lambda_{effective}$) is well correlated with temperature variation. During stage 1 (hardening) dislocations’ density increases which reduces thermal conductivity. During the softening region, the annihilation of dislocations improves thermal conductivity before stage 3 starts. During stage 3 as cyclic stabilization, dislocation structures are self-organized in the form of PSBs and walls are fully developed and extend all over the testing sample as fatigue cycles continue during stage 3. Therefore, the second reduction in thermal conductivity or temperature change is rational. The final increase in $\lambda_{effective}$ or temperature change pertains to microcracks formation (can be called secondary structures). Microcracks’ formation results in new surfaces creation and releasing more heat from the test sample. The above experimental results of Huang et al. work confirm the correlation among self-organization emergence, dissipated heat, stored energy, and dislocation density.

##### 3.3.2. Acoustic Emission

**Figure 9:**
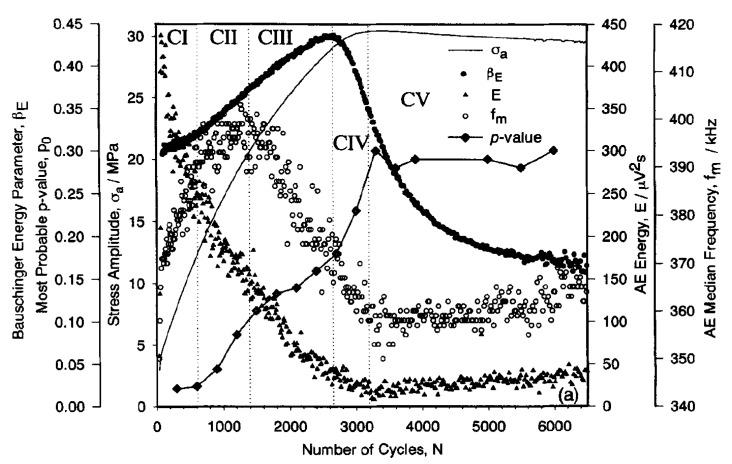
Comparison of cyclic hardening and acoustic emission (AE) signals for copper single crystal [171]. AE features are energy, median frequency, and pulse position. Bauschinger energy parameter is related to the area of the hysteresis loop (with permission).

Monitoring temporal and spatial evolution of acoustic emission (AE) and sound arising from dislocations interactions is a powerful technique to macroscopically witness self-organization and patterning emergence during fatigue of materials. AE signals carry unique information regarding structural change, movement, and activities of defects and their interactions once subjected to repeated loading [171]. Among the AE published works related to self-organization pattern formation and acoustic entropy available in the literature [171,172,173,174,175,176,177,178,179,180,181,182,183,184,185,186], the works of Vinogradov and co-workers are very interesting in terms of connecting AE features to dislocation dynamics and self-organization [171,172]. Their fatigue tests on copper single crystals and polycrystals showed a strong correlation between AE features and self-organization emergence. As an example, Figure 9 compares intercorrelation between AE features (energy, median frequency, and pulse position parameter) and cyclic stress-strain curve for copper single crystals [171,172]. It is seen that similar to stored energy and temperature evolution, AE features correlate well with dislocation activities and structural transformations within the crystal.

As seen in Figure 9, the stress amplitude increases along with cyclic hardening before saturation is achieved at 3200 cycles. As pointed out by Vinogradov et al. [171], initially, AE energy rises to its maximum for a few cycles due to easy-glide in dislocation activities and then tends to descend rapidly with increasing hardening. As cyclic load proceeds, dislocation density increases resulting in the reduction of dislocations mean free path and consequently reduction in AE energy [171,179]. At the onset of transition to saturated stress, AE energy attains its minimum values. The emergence of PSBs and self-organization corresponds to the low-frequency AE spectrum. The reason is attributed to the existence of PSBs which have lower dislocation density, higher dislocation movement, longer glide distance, and softer behavior than matrix [105,187]. In the saturation regime, gradual increase in AE energy is noticeable [171,172]. After saturation, PSBs tend to harden gradually [188] leading to a slight increase in the dislocation density and movement. Therefore, AE energy tends to gradually increase after 3200 cycles [171]. Other AE features are also presented in Figure 9. The detailed explanations of other AE features (i.e., median frequency; *f*_m_) are referred to the work of Vinogradov et al. [171].

##### 3.3.3. Self-Organization through External Elements during Fatigue

**Figure 10:**
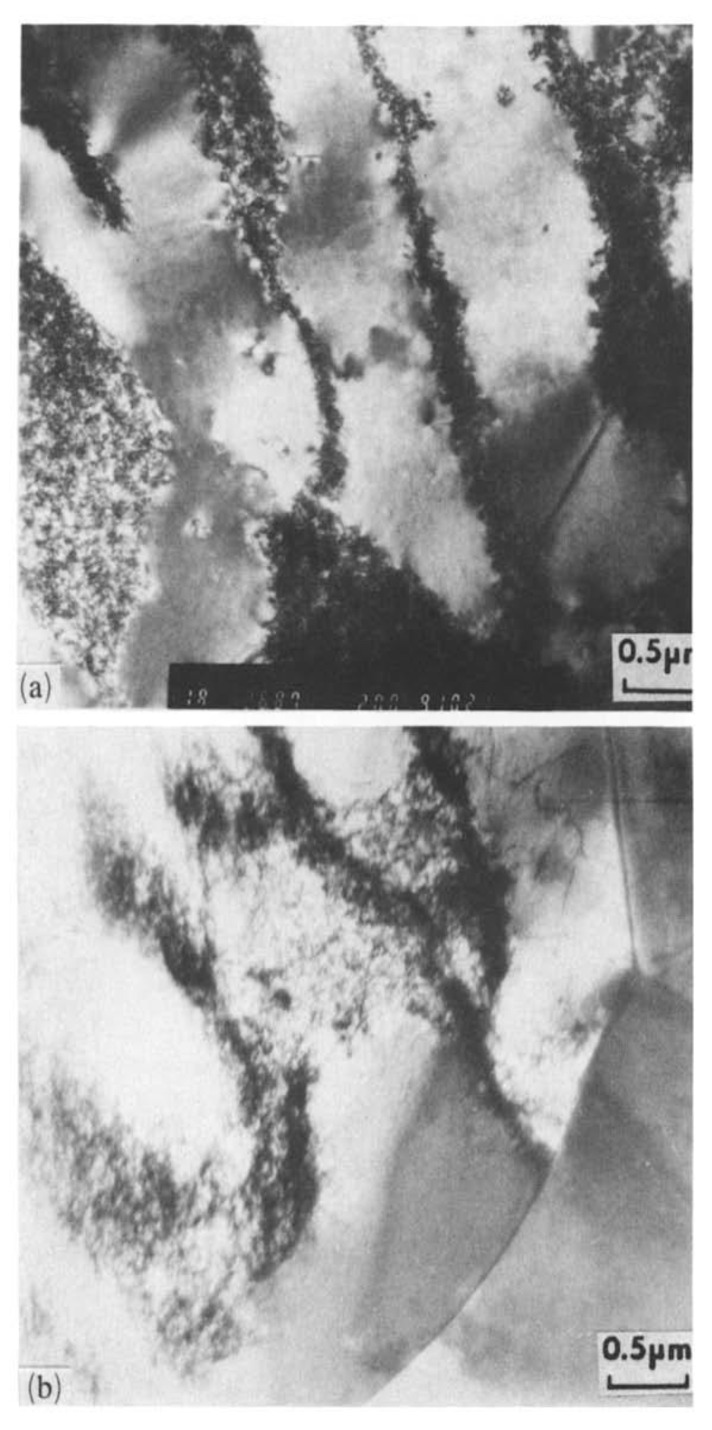
TEM image of dislocation arrangement in polycrystalline copper at the stress level of 102 Mpa (**a**) without current pulse and fatigue failure life at 8 × 10^5^ cycles, (**b**) with current pulse and fatigue failure life at 2.1 × 10^6^ cycles [189] (with permission).

As reported by some researchers, fatigue life can be improved by applying external elements such as electric current, magnetic field, and surface cooling [189,190,191,192,193,194,195,196,197,198,199,200,201,202,203,204,205,206]. Conrad et al. experimentally investigated the effect of electric current pulses on the fatigue life of polycrystalline copper in a series of rotating-bending fatigue tests [189]. They observed that with current pulse fatigue life can be extended by a factor of 2–3 especially for low-stress levels. They suggested that the increased number of cycles for crack initiation with electropulsing is related to the reduction in the spacing and width of PSBs (i.e., an increased homogenization of slip bands). They also postulated that the increased homogenization of slip might be attributed to the increase in dislocation mobility due to the interaction between the active dislocations and the drift electrons held by the current, or rather from electromigration effects [189,207,208]. Figure 10 shows TEM image of dislocation arrangement in polycrystalline copper at the stress level of 102 Mpa without and with current pulse [189]. It could be seen that with current pulse, dislocations can be less tightly bound within walls and cell boundaries postulating lower dislocation density [189]. As pointed out by Khonsari and Amiri, homogenization of slip bands is analogous to the notion of self-organization [205].

Magnetic field as another external element can prolong fatigue life as reported by researchers [192]. Zhao et al. [192] applied an alternating magnetic field on A3 steel and observed substantial improvement in fatigue life. They ascribed this enhancement to the effect of pumping negative entropy into an open thermodynamic system and formation of dissipative structures during fatigue without performing a detailed thermodynamic analysis. Celik et al. investigated the effect of the magnetic field with different intensities on the fatigue life of AISI 4140 steel. They found out if the magnetic field is applied at the stage of fatigue crack initiation, the fatigue life is improved due to postponing time required for slip bands’ formation. Magnetic field energizes movement and energy of dislocations by assisting them to overcome obstacles, and consequently, time required for slip bands, formation is prolonged [194,195]. From the thermodynamic point of view discussed in previous sections, the effect of the magnetic field could have a temporal effect on the formation of dissipative structures, and help the self-organization process, and slip bands nucleation and developments undergo more smooth evolutionary process. Hence, its effect delays the process of microcracks formation.

In an interesting work, Amiri and Khonsari studied the effect of surface-cooling on the fatigue life of Stainless Steel 304L and Steel 4145 specimens undergoing low-to-intermediate-cycle fatigue through a series of rotating-bending tests [204,205]. They observed a significant improvement in fatigue life with the surface-cooling and postulated that the notion of self-organization and dissipative structures formation might be responsible for improved fatigue. Further details are referred to the works of Khonsari and Amiri [204,205].

For a thermodynamic system far from equilibrium, fluctuations emitting from either internal (i.e., dislocation density) or external (i.e., electric or magnetic current) sources can drive the system into a new stable regime. To obtain insight into how the evolutionary process of dissipative structures formation during fatigue can be influenced and how fatigue degradation can be postponed by applying external elements such as electric or magnetic field, we elaborate the relationship between dislocation density and applied electric current within a simple example. Assuming the entropy generation rate can be expressed as:

(14) $$\frac{dS_{i}}{dt}=X_{1}J_{1}+X_{2}J_{2}$$

The first term on the right-hand side is the force and flux of dislocation density and the second term represents the force and flux due to applied electric current as follows:

(15) $$X_{1}J_{1}=\left(\frac{AGb^{2}}{T}\right)\left(\dot{\rho }\right)\;;\;X_{2}J_{2}=\left(\frac{V}{T}\right)\left(J_{e}\right)$$

where *A* is a constant, *G* is the shear modulus, *b* represents burger vectors, $\dot{\rho }$ is total dislocation density rate, and *T* is temperature. *V* represents applied external voltage and $J_{e}$ is the flow of electric current. Assuming that applied external voltage is constant and shear modulus and temperature change with respect to electric current are insignificant, one can minimize entropy generation with respect to electric current.

(16) $$\left(\frac{d}{dJ_{e}}\right)dS_{i}=0$$

(17) $$\left(\frac{d}{dJ_{e}}\right)dS_{i}=\left(\frac{AGb^{2}}{T}\right)\frac{d\dot{\rho }}{dJ_{e}}+\frac{V}{T\;}\;;\;\left(\frac{AGb^{2}}{T}\right)\frac{d\dot{\rho }}{dJ_{e}}=-\frac{V}{T}$$

(18) $$\dot{\rho }={\dot{\rho }}_{0}-\frac{V}{AGb^{2}}J_{e}$$

where ${\dot{\rho }}_{0}$ is the dislocation density rate without applying current.

Equation (18) with simplified assumptions shows that theoretically an increase in electric current slows down overall dislocation density rate resulting in minimizing entropy and degradation rate. The electric current influences the dislocation generation and annihilation rate which are two competing mechanisms. It is noted that if the temperature change is significant by electric current the above assumptions lead to Equation (18) are ruled out and the effect of electric current on constants *T*, *G,* and *A* should be taken into account during the derivative process. This analysis describes that it is possible to reduce the fatigue degradation process using external processes such as electric current which spatiotemporally alters the completion of dissipative structures formation.

## 4. Discussion on the Stability of Self-Organized Structures

The formation of dissipative structures and the self-organization process during fatigue were discussed in the previous sections with some examples. In what follows the author shed light on simple thermodynamic stability analysis regarding presented fatigue-related examples.

According to Prigogine [4,5], for a thermodynamic system far from equilibrium, turning points, and bifurcation points can be characterized as “birth of order out of chaos through fluctuations”. Organized patterns are formed within length scales ranging from nanometers to millimeter level, and time scales ranging from picoseconds to a few hours. Phenomenon accompanying self-organization (i.e., here spontaneous formation of veins, PSBs, cells, labyrinth) process is the loss of stability of the thermodynamic branch. Therefore, how far from the bifurcation point self-organized structures are stable depends on time and length scale. It might vary from nano-second to some hours. As the evolution process continues in a thermodynamic system, interaction, multiplication, aggregation, and annihilation of control parameters (i.e., dislocations) drive thermodynamic system to a new level of complexity and orderliness.

For example, previously presented single crystal CSS curve and appearance/disappearance of plateau clearly shows that whether self-organized PSBs can remain stable during cycling strongly depends on driving force, and type and orientation of crystal. For some slip modes (i.e., [011] slip mode) of copper single crystal under low or intermediate plastic strain with a clear plateau regime, PSBs-ladder structure can stay stable for many cycles far away from the bifurcation point (initiation of saturation regime) before next instability starts (appearance of labyrinths, cells, or microcracks). While, for other slip modes or higher plastic strain, PSBs are not either formed or if formed, their duration with increasing cycles might be short and quickly followed by another instability or predominant secondary slip system. Therefore, existing stable saturation regime or non-existing saturation region is strongly dependent on the modes and intensities of dislocation interactions among slip systems active in the crystals, and different slip deformation characteristics (primary and secondary slip systems) as well as the applied driving force.

**Figure 11:**
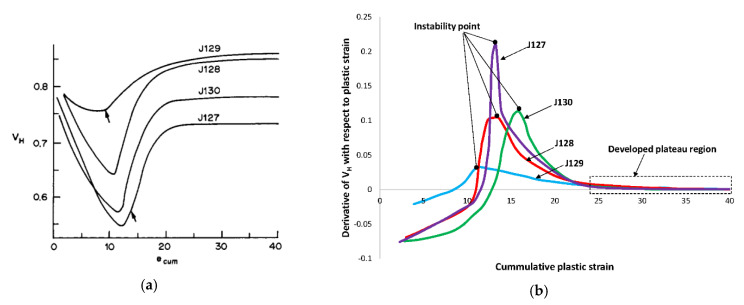
(**a**) Variation of V_H_ with cumulative strain for different plastic shear strain. Arrows show the first appearance of PSBs on surfaces [106] (with permission); (**b**) the derivative of V_H_ with respect to cumulative plastic strain. Plastic shear strain amplitudes for J127, J130, J128, J129 specimen are 0.78, 1.45, 1.95, 3.02 (×10^−3^).

To elaborate more on the importance of stability and PSBs, change of the normalized hysteresis loop area of fatigued copper single crystal oriented for single slip as a function of cumulative strain at low and high plastic strain is shown in Figure 11a as reported by Jin [106]. Solid lines are pertained to test specimens at the different plastic strain level. The abscissa (*ε*_cum_) is cumulative strain while the ordinate is a dimensionless area of hysteresis (*V_H_*). *V_H_* is defined as the ratio of the area enclosed by the loop divided by the area of the circumscribing rectangle [104]. *V_H_* initially decreases passing through a minimum, then increases followed by a saturation or quasi-saturation regime. Instability point is clear as sharp turning points on the curves. Measured hysteresis loops reflect drastic structural change in turning points where PSBs start to nucleate by veins’ destruction. Jin observed that bifurcation points change from a sharp behavior for low amplitude plastic strains (i.e., J127 with e_p_ = 0.78 × 10^−3^) towards more gradual behavior at high amplitude plastic strains (i.e., J129 with e_p_ = 3.02 × 10^−3^). Volume fraction of veins at low plastic strain are much higher than that of veins at a high plastic strain. They argued that for low plastic shear strains, dominant mechanism of PSB formation is the destruction of high volume fraction veins leading to dramatic structural change. On the other hand, for high plastic strain amplitude, the low volume fraction of veins leads to more gradual PSBs walls formation alongside matrix veins [106]. Further discussion is referred to the work of Jin [106], Kuhlmann and Laird [209,210], Winter [105], Mecke et al. [211], and Tabata et al. [212].

In addition, shown in Figure 11b is the derivative of the curves constructed from Figure 11a. After instabilities take place and dissipative structures are formed, the rate of change in the hysteresis loop area is roughly invariant confirming that input mechanical work is not destructive and is not stored in the crystals. This can be justifiable with the work of Seeger [133,135] on stored energy as an indication of self-organization.

**Figure 12:**
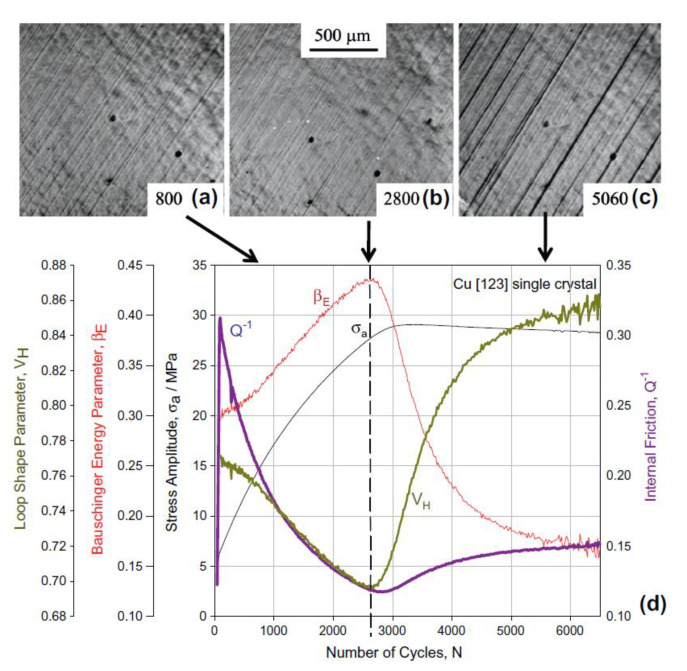
(**a**–**c**) typical surface morphology evolution versus fatigued; (**d**) evolution of cyclic stress (σ_a_), hysteresis loop shape parameter V_H_, internal friction Q^−1^, and energy parameter β_E_ for copper single crystal oriented for easy glide. Dashed line corresponds to bifurcation point as the onset of vein rupture and PSBs emergence. The figure is quoted from [172] (with permission).

Another compelling example to show the relationship between instabilities and patterning emergence is presented in Figure 12 reported by Vingradov and Yashinkov [172] for copper single crystal subjected to cyclic loading. The authors’ experimental finding reveals a strong correlation between the time of instability (onset of the emergence of patterning and self-organization) and evolution of measurable quantities such as AE, internal friction (IF), Bauschinger energy parameter ($\beta_{E}$), hysteresis loop parameter (V_H_). It is clear in Figure 12d that the onset of self-organization and PSBs formations are well-correlated with the minima of V_H_ and IF evolution and maxima of $\beta_{E}$. IF is the ratio of hysteresis loop to the total mechanical work done on the system. Bauschinger energy parameter is a measure of hysteresis loop shape and is interconnected to *V_H_* ($V_{H}=1/\left(1+\beta_{E}\right)$). All measurable quantities, as well as AE presented in Figure 12, confirm the number of cycles of 3200 is roughly the onset of instability toward PSBs formation and pattern formation.

The stability of the non-equilibrium thermodynamic system can be characterized using Lyapunov’s function. Once a non-equilibrium thermodynamic process passes through an instability point, process of self-organization begins [4]. Above experimental evidence of fatigued copper single crystal revealed self-organization and formation of dissipative structures initiate roughly around saturation regime once instability initiates (disintegration of veins). In what follows the author seeks to show that during fatigue process self-organization can be achieved through the application of the excess entropy production and Lyapunov’s function.

During plastic deformation, entropy production associated with dislocation generation, dislocation annihilation, and dislocation glide is expressed as follows [213,214,215].

(19) $$dS_{i}=\frac{dW_{p}}{T}+\frac{dW_{g}}{T}+\frac{dW_{a}}{T}$$

where *T* is the absolute temperature. $dW_{p}$, $dW_{g}$, and $dW_{a}$ represent energy dissipations through dislocation generation, glide, and annihilation.

Dissipative energy of dislocation generation can be expressed as a function of the density of dislocation generation, $d\rho^{+}$ during time interval *dt* [213,214,215,216].

(20) $$dW_{p}=\frac{Gb^{2}}{2}d\rho^{+}$$

where *G* is the shear modulus and *b* is the magnitude of the Burgers vector.

Dissipative energy of dislocation annihilation is proportional to the density of dislocation annihilation, $d\rho^{-}$ during time interval *dt* as follows.

(21) $$dW_{p}=\frac{Gb^{2}}{2}d\rho^{-}$$

The energy dissipated through dislocation glide responsible for moving newly generated dislocations is expressed as [213,214,215]:

(22) $$dW_{p}=\tau bld\rho^{+}$$

where τ is stress and a function of average total dislocation density (ρ) via [217,218]:

(23) $$\tau =\tau_{0}+\alpha Gb\sqrt{\rho }$$

where $\tau_{0}$ is the representation of lattice resistance and solid solution and can be neglected for pure FCC single crystal [219]. Parameter α is constant and depends on temperature *T* and strain rate $\dot{\gamma }$. Incremental change of average dislocation density dρ is the difference between dislocation generation and annihilation variations ($d\rho =d\rho^{+}-d\rho^{-}$).

The entropy production can be expressed as the sum of the products of generalized forces and their corresponding fluxes of irreversible processes [2,4].

(24) $$\frac{dS_{i}}{dt}=X_{p}J_{p}+X_{g}J_{g}+X_{a}J_{a}$$

where $X_{p}$ is thermodynamic force due to dislocation generation ($X_{p}=\frac{dW_{p}}{Tdt}$), $X_{g}$ represents thermodynamic force due to dislocation glide ($X_{g}=\frac{dW_{g}}{Tdt}$), and $X_{a}$ is thermodynamic force due to dislocation annihilation ($X_{a}=\frac{dW_{a}}{Tdt}$).

Using the definition of excess entropy production (δXδJ) and according to Equation (11) the stability of non-equilibrium process can be expressed by following in-equality.

(25) $$\frac{1}{2}\frac{\partial \left(\delta^{2}S_{i}\right)}{\partial t}=\delta X_{p}\delta J_{p}+\delta X_{g}\delta J_{g}+\delta X_{a}\delta J_{a}\geq 0$$

where $\delta X_{i}$ and $\delta J_{i}$(*i*= p, g, a) are the deviation of thermodynamic forces and fluxes from a stationary state. If the above inequality is satisfied, then the given state is stable. Otherwise, the stability condition is violated, the fatigued open system is likely to enter into a self-organized regime with reduced entropy.

Entropy production $dS_{i}\;$can take the following form after combining Equations (20)–(22) as

(26) $$dS_{i}=\left(1+2\alpha \right)\frac{Gb^{2}}{2T}d\rho +\left(2+2\alpha \right)\frac{Gb^{2}}{2T}d\rho^{-}$$

Let us assume that during cyclic plastic deformation, dislocation generation, glide, and annihilation are the dominant energy dissipation process. It is assumed that thermodynamic force, *X(T,* $\dot{\gamma }$*)* and flux, *J (T,* $\dot{\gamma }$*)* are only a function of temperature and shear strain rate ($\dot{\gamma }$). For simplicity, the dependency of *X* and *J* to other internal variables is not considered.

Excessive entropy production for Equation (24) has the following forms:

As the strain rate ( $\dot{\gamma }$) varies:

(27) $$\begin{array}{ll} \frac{1}{2}\frac{\partial \left(\delta^{2}S_{i}\right)}{\partial t}=\frac{Gb^{2}}{T} & \frac{\partial \alpha }{\partial \dot{\gamma }}\frac{\partial \dot{\rho }}{\partial \dot{\gamma }}{\left(\delta \dot{\gamma }\right)}^{2}+\frac{Gb^{2}}{T}\frac{\partial \alpha }{\partial \dot{\gamma }}\frac{\partial {\dot{\rho }}^{-}}{\partial \dot{\gamma }}{\left(\delta \dot{\gamma }\right)}^{2}\\ & =\frac{Gb^{2}}{T}\frac{\partial \alpha }{\partial \dot{\gamma }}\left(\frac{\partial \dot{\rho }}{\partial \dot{\gamma }}+\frac{\partial {\dot{\rho }}^{-}}{\partial \dot{\gamma }}\right){\left(\delta \dot{\gamma }\right)}^{2}=\frac{Gb^{2}}{T}\frac{\partial \alpha }{\partial \dot{\gamma }}\frac{\partial {\dot{\rho }}^{+}}{\partial \dot{\gamma }}{\left(\delta \dot{\gamma }\right)}^{2} \end{array}$$

The stability condition is violated if $\frac{\partial \alpha }{\partial \dot{\gamma }}$ and $\left(\frac{\partial \rho^{}}{\partial \gamma }+\frac{\partial \rho^{-}}{\partial \gamma }\right)$ or $\frac{\partial \rho^{+}}{\partial \gamma }$ have different signs. If $\frac{\partial \alpha }{\partial \dot{\gamma }}>0$, the stability condition is determined by the competition between the rate of dislocation generation and annihilation density with respect to shear strain. It is stated that formation of PSBs is initiated by the disintegration of veins or loop patches. It is relevant to connect excess entropy generation terms to dislocation evolution inside the veins. Any perturbation in dislocation generation or annihilation which makes $\left(\frac{\partial \rho^{}}{\partial \gamma }+\frac{\partial \rho^{-}}{\partial \gamma }\right)<0$ while $\frac{\partial \alpha }{\partial \dot{\gamma }}>0$, takes non-equilibrium thermodynamic branch toward a newly organized branch.

Similar to strain rate change, as the temperature (*T*) varies:

(28) $$\frac{1}{2}\frac{\partial \left(\delta^{2}S_{i}\right)}{\partial t}=\frac{Gb^{2}}{2T}\left(\frac{2\partial \alpha }{\partial T}-\frac{1+2\alpha }{T}\right)\frac{\partial \dot{\rho }}{\partial T}{\left(\delta T\right)}^{2}+\frac{Gb^{2}}{2T}\left(\frac{\partial \alpha }{\partial T}-\frac{2+2\alpha }{T}\right)\frac{\partial {\dot{\rho }}^{-}}{\partial T}{\left(\delta T\right)}^{2}\;=\frac{Gb^{2}}{T}\left\{\frac{\partial \alpha }{\partial T}\frac{\partial {\dot{\rho }}^{+}}{\partial T}-\frac{1}{2T}\left[\left(1+2\alpha \right)\frac{\partial {\dot{\rho }}^{+}}{\partial T}+\frac{\partial {\dot{\rho }}^{-}}{\partial T}\right]\right\}{\left(\delta T\right)}^{2}$$

The instability condition is decided by the signs of $\frac{\partial \alpha }{\partial T}$, $\frac{\partial {\dot{\rho }}^{+}}{\partial T}$, and $\frac{\partial {\dot{\rho }}^{-}}{\partial T}$ and how dislocations density changes with temperature in the above equation. Any perturbation in the dislocation density leading to the negativity of the above expression results in the bifurcation from thermodynamic branch and the emergence of patterns. Combining Equations (27) and (28) leads to the simultaneous study of temperature and strain rate effect on the excess entropy production and instability criterion.

To further detail how instability emerges through above mentioned excess entropy inequality, it is required to visit the different mechanisms triggering vein disintegrations and control parameters influencing the rate of dislocation density concerning temperature and plastic strain within a statistical approach. Control parameters such as dislocations velocity, the oscillation of dislocation density, the activation energy of dislocation climbs, types of dislocation (edge, screw, etc.), characteristic of perturbed dislocation density wave (i.e., frequency, components of wave vector, etc.) might be considered for better comprehensive analysis of above excess entropy production inequality. The works reported in [92,93,95,96,97,99,103,105,106,109,116,210,212,220,221,222,223,224,225] contain useful and very interesting discussions related to instability analysis and vein breaching mechanisms.

Self-organized structures and orderliness emerging after instability point reduce entropy generation locally and temporally and can be beneficial for fatigue process. During fatigue as a non-equilibrium irreversible process, a fatigued system has the potential to stay stable for many cycles far away from the bifurcation point before nucleation of a noticeable crack. Consequently, fatigue damage accumulation slows down after pattern and self-organization emergence. The concept of minimum entropy generation at stationary non-equilibrium processes can be very helpful in the design of fatigue resistant materials if the notion is applicable. It is noted that the principle of minimum entropy production of non-equilibrium processes proposed by Prigogine [4,6] is formulated during self-organization process by [87,88] states that entropy generation is lowered compared prior to self-organization process. Future developments toward inventing fatigue resistance metals should take into account the in-depth knowledge of self-organization and dissipative structures’ formation within the framework of non-equilibrium thermodynamics.

An important path in material science is to reduce localization of the deformation using material hardening mechanisms and pattern formation. If correctly recognized and applied, the concept of self-organization and dissipative structures formation can be used for rational selection and the development of new metals with specific properties and favorably crystallographic orientations to facilitate patterning and orderliness while subjected to external loading. A recent impressive work of Pan et al. [226] shows how the concept of patterning at the nanoscale can lead to design better fatigue resistant metals. Similar works related to highly oriented nanotwinned metals for damage tolerant and fatigue-resistance materials can be referred to the work of [13,227,228,229,230].

The concept of patterning can justify the existence of a material property at different length scales for damage nucleation criterion. Since the stored energy of self-organized dislocations roughly invariant before cracks nucleate, stored energy concept can be used in a computational framework as a criterion for fatigue damage nucleation. Using the concept that the balance between stored energy and dissipated energy is achieved after the establishment of dislocation structures, Wan et al. found that critical stored energy is a unique material property for fatigue crack nucleation [137,138]. Others also used the stored energy and entropy related criterion for fatigue crack nucleation and life scatter predictions [139,231,232,233,234].

## 5. Conclusions

The main theme of this review was the application of self-organization notion and to elucidate the importance of self-organization, patterning, and orderliness in the material science field especially fatigued metallic crystals. This review tried to provide vital and important aspects and evidence of the self-organization phenomenon in cyclic deformation of some single metallic crystals. The aforementioned compelling examples obtained from literature during fatigue process revealed that at the emergence of PSBs or appearance of plateau regime in CSS curve, a dynamic equilibrium exists between dislocation multiplication and annihilation and the damage or stored energy reaches a minimum level. It was seen that AE signals and temperature profile carry unique information regarding structural change, movement, and activities of dislocations and their interactions once subjected to repeated loading.

Plastic deformation is an irreversible process driving crystalline materials into a non-equilibrium state and possibly resulting in the formation of pattern and self-organized microstructures. Macroscale mechanical performance of materials after going through microstructural change and the formation of dissipative structures can be either detrimental or beneficial. In-depth understanding of the conditions leading to the dissipative structures formation and self-organized structures might thus lead us toward improving and designing novel materials with exceptional properties.

Future success in developing novel materials is highly tightened to our understanding of non-equilibrium thermodynamics, self-organization, thermodynamic instabilities, and fluctuations from a fundamental point of view. It requires our understanding of how to engineer and control the process of self-organization, pattern formations, and instabilities during any physico-chemical–mechanical process.
